# Global research trend of Herpes simplex keratitis: a bibliometric analysis and visualization from 1941 to 2024

**DOI:** 10.3389/fmed.2025.1526116

**Published:** 2025-03-19

**Authors:** Ke Song, Shujiao Li, Jian Liu, Zefeng Kang

**Affiliations:** China Academy of Traditional Chinese Medicine Hospital of Ophthalmology, Beijing, China

**Keywords:** bibliometric analysis, Herpes simplex keratitis, VOSviewer, CiteSpace, visualization

## Abstract

**Objective:**

Herpes simplex keratitis (HSK), caused by the herpes simplex virus (HSV), is a leading cause of infectious blindness worldwide. This study aims to explore the research trends, key contributors, and emerging areas of focus in HSK research through bibliometric analysis.

**Methods:**

Publications related to HSK from 1941 to 2024 were retrieved from the Web of Science Core Collection (WoSCC). Bibliometric and visual analyses were conducted using VOSviewer, CiteSpace, and R 4.3.3.

**Results:**

A total of 1,076 publications on HSK were identified. The top three contributing countries were the United States (267 papers), China (99), and Japan (64). Harvard University was the leading institution with 75 publications, while the *American Journal of Ophthalmology* emerged as the most influential journal, boasting an h-index of 29. Kaufman, HE, was the most cited author, with 1,988 citations. The top three keywords were “infection” (82), “stromal keratitis” (73), and “penetrating keratoplasty” (62). Burst keyword analysis indicated a growing interest in terms such as “outcome” and “ultraviolet A” since 2018.

**Conclusion:**

This bibliometric analysis underscores two primary research areas in HSK: the clinical management of stromal keratitis and infection, as well as the mechanisms of HSK recurrence, which include strategies for preventing reactivation and managing immune rejection. Future research is anticipated to focus on innovative treatments, particularly ultraviolet A therapy.

## Introduction

Herpes simplex keratitis (HSK) is a corneal inflammation and one of the leading infectious causes of blindness worldwide. Its annual incidence is estimated at 10–20 cases per 100,000 people (1, 2). Reactivation, often triggered by factors such as immunosuppression or stress, leads to recurrent episodes, causing chronic corneal inflammation and permanent corneal damage (3). The pathogenesis of HSK is complex, involving viral replication, abnormal host immune responses, and neuroinflammatory processes (4). Recurrent episodes can lead to corneal scarring, ulceration, and blindness, significantly impairing patients’ quality of life (5). The high recurrence rate further complicates management and treatment.

Given the severe consequences of HSK, timely and effective intervention is essential to prevent vision loss (6, 7). Antiviral medications, such as acyclovir and valacyclovir, are the primary treatment options, effectively inhibiting viral replication (8, 9). Additionally, topical corticosteroids are used to control inflammation, and immunomodulators like cyclosporine A help prevent recurrences (3). In recent years, corneal transplantation has become an option for advanced cases, though treatment outcomes vary depending on the patient’s immune response (10, 11). Despite the availability of diverse treatment options, further optimization is needed to improve efficacy and reduce recurrence (1).

Bibliometrics is a tool for quantitatively analyzing scientific literature to reveal trends in discipline development, research hotspots, and scientific output (12). Unlike traditional reviews, bibliometric analysis processes large volumes of literature, providing a more comprehensive and systematic overview (13). Through the examination of burst keywords and citation networks, bibliometric techniques can reveal emerging topics that may not yet be widely recognized but have the potential to influence clinical practice in the near future (14). The insights gained from bibliometric analysis can help clinicians and researchers identify high-impact studies, key collaborative networks, and areas that are underexplored, ultimately supporting evidence-based decision-making and resource allocation. Given the importance of understanding the landscape of HSK research, it is necessary to conduct bibliometric analysis to explore its research hotspots and trends. However, there is currently a lack of such analysis specifically focused on HSK. This study aims to systematically analyze the literature on HSK using bibliometric methods, identifying research hotspots, key scholars, and institutions, while exploring the knowledge structure and development trends in this field.

## Materials and methods

### Literature search

A comprehensive literature search was conducted using the Web of Science Core Collection (WoSCC), an interdisciplinary database widely used in bibliometric research, to identify relevant studies on HSK. The search was performed on June 24, 2024, ensuring the inclusion of the most up-to-date publications. The search query used was: [TS = (“Herpes Simplex Keratitis” OR “Herpes Simplex Virus Keratitis” OR “Herpes Simplex Viral Keratitis” OR “Herpetic Keratitis” OR “Keratitis, Herpes Simplex”)]. The search was restricted to English-language articles.

### Statistical analysis and visualization

Microsoft Excel was utilized to organize and analyze the retrieved data, calculating key bibliometric indicators such as annual publication counts, citation frequencies, average citation rates, and information on journals, impact factors, publication countries/regions, institutions, and authors. Three bibliometric tools were employed for data visualization: VOSviewer (version 1.6.20), CiteSpace (version 6.3.R1), and R 4.3.3. VOSviewer was used to map institutional collaborations, author collaborations, co-authorship networks, citations, and co-citations (15), as well as for keyword co-occurrence analysis to reveal complex academic relationships and identify emerging research themes. CiteSpace was employed to detect keyword bursts, with the parameters set for time slicing from January 1994 to July 2024 in 1-year intervals (16). The node type was set to keywords, with a top N threshold of 5 for each time slice. Pruning was conducted using the pathfinder and merged network methods, and a timeline graph was generated to display keyword trends in HSK research. In the visual representations, node sizes corresponded to the number of publications, line thickness indicated the strength of connections, and node colors represented different clusters or time periods. To quantify the academic impact, metrics such as the H-index, g-index, and m-index were applied, as outlined in existing literature (17–19). Additionally, Journal Citation Reports (JCR) quartiles and Impact Factors (IF) in 2023 were used to assess the significance and influence of journals publishing HSK-related studies (20).

## Results

### An overview of publications

**Figure 1:**
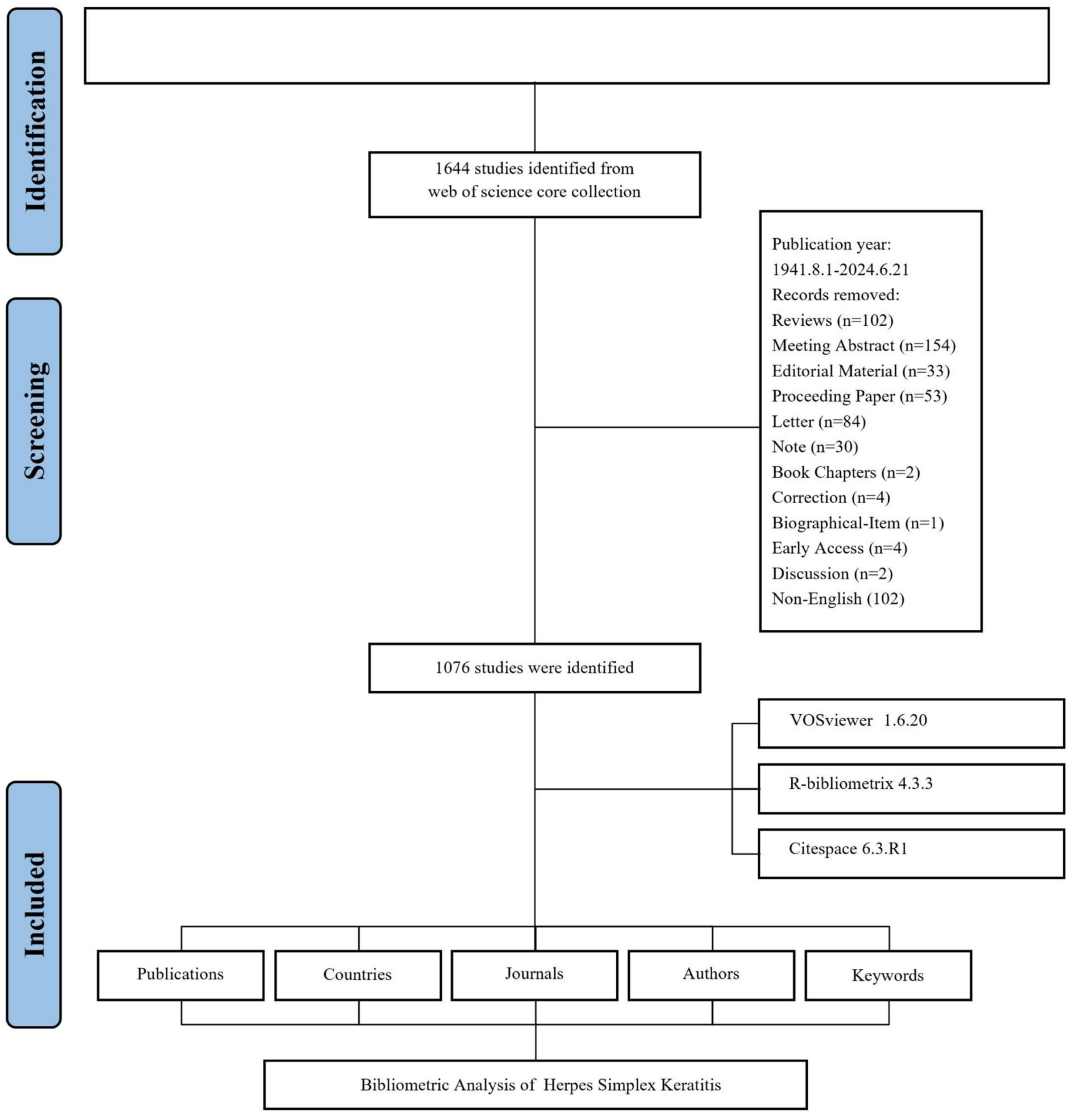
Flowchart of the literature screening process.

**Figure 2:**
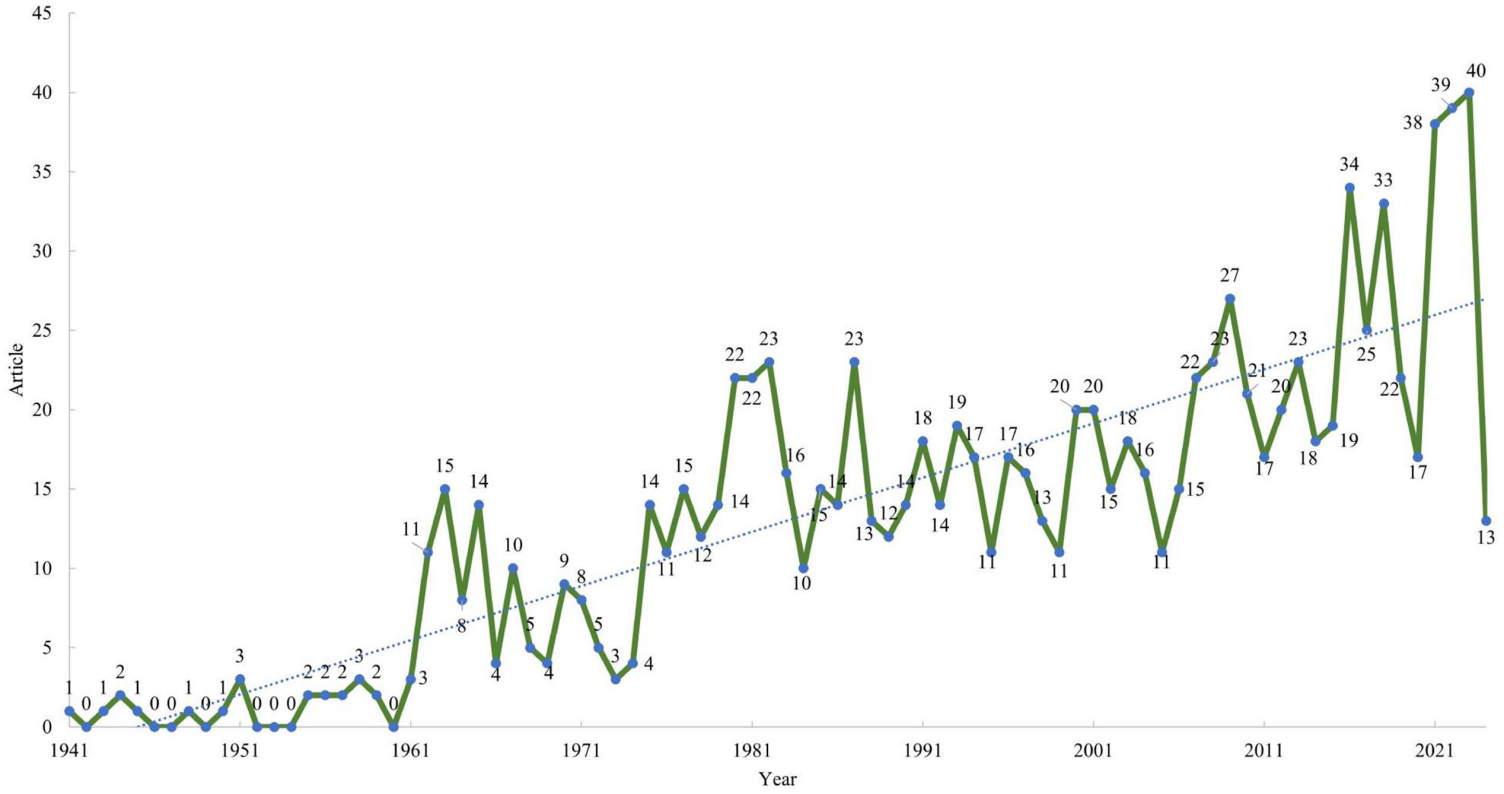
Annual number of publications on Herpes simplex keratitis. Data sourced from Web of Science Core Collection (WoSCC).

This study analyzed a total of 1,076 publications, with the data screening process depicted in Figure 1. The number of publications on HSK research per year from 1941 to 2024 is illustrated in Figure 2. Initially, publication numbers were low and relatively stable, with fewer than 10 articles published per year until the early 1960s. A notable rise occurred around 1964, peaking at 15 publications. Over the subsequent decades, publication counts fluctuated between 10 and 23 articles annually. However, a significant upward trend began in the 2000s, with 27 articles published in 2009, increasing to 39 in 2022, and peaking at 40 in 2023.

### Analysis of countries

**Figure 3:**
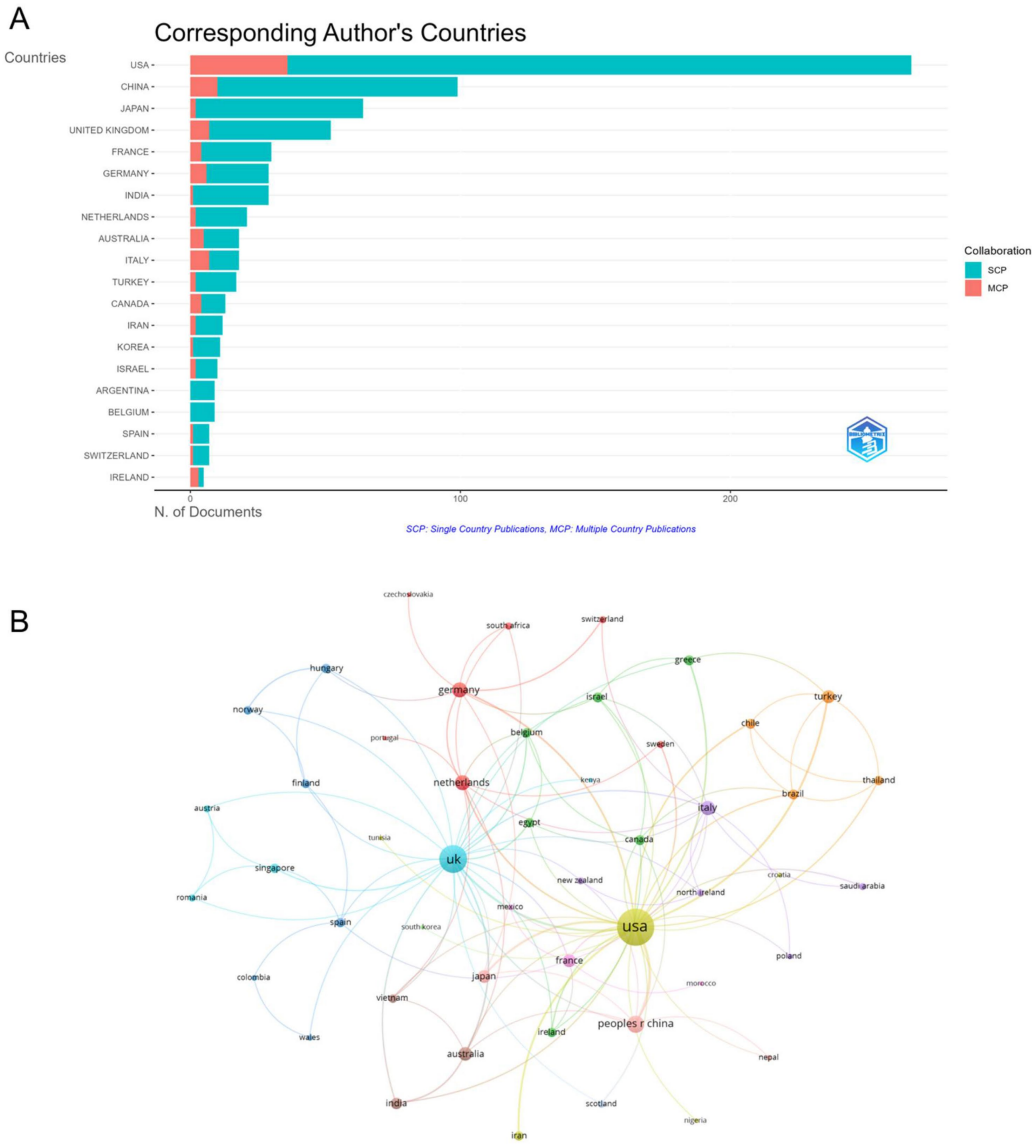
Analysis of countries. **(A)** Distribution of publications by corresponding authors by country, derived using the Bibliometrix package in R 4.3.3. **(B)** Visualization map depicting collaboration among different countries using VOSviewer.

The top three contributing countries in the field of HSK research are the United States (267 articles), China (99 articles), and Japan (64 articles). Of the top 10 countries, the Netherlands, Australia, and Germany have the highest average article citations, with scores of 45.6, 38.7, and 28.7, respectively. While most publications were single-country outputs, international collaborations also played a significant role. The Multiple Country Publications (MCP) Ratio was highest for Ireland (0.6), followed by Italy (0.389) and Canada (0.308) (Table 1 and Figure 3A). The USA leads with the highest number of total citations (7,900), followed by the United Kingdom (1,329) and Japan (1,073) (Figure 3B).

**Table 1:** Publication and citation profiles of leading countries.

Country	Articles	Freq	MCP_Ratio	TC	TC_RANK	TP	TP_RANK	Average article citations
USA	267	0.248	0.135	7,900	1	599	1	29.6
China	99	0.092	0.101	806	6	286	2	8.1
Japan	64	0.059	0.031	1,073	3	191	3	16.8
United Kingdom	52	0.048	0.135	1,329	2	157	4	25.6
France	30	0.028	0.133	701	7	129	5	23.4
Germany	29	0.027	0.207	832	5	66	8	28.7
India	29	0.027	0.034	405	9	71	7	14
Netherlands	21	0.02	0.095	957	4	75	6	45.6
Australia	18	0.017	0.278	696	8	59	10	38.7
Italy	18	0.017	0.389	344	11	66	9	19.1
Turkey	17	0.016	0.118	247	13	28	15	14.5
Canada	13	0.012	0.308	191	15	34	11	14.7
Iran	12	0.011	0.167	131	17	34	12	10.9
Korea	11	0.01	0.091	175	16	26	16	15.9
Israel	10	0.009	0.2	106	20	28	14	10.6
Argentina	9	0.008	0	365	10	22	18	40.6
Belgium	9	0.008	0	283	12	31	13	31.4
Spain	7	0.007	0.143	78	21	17	21	11.1
Switzerland	7	0.007	0.143	118	19	20	19	16.9
Ireland	5	0.005	0.6	66	22	24	17	13.2

Articles, Publications of Corresponding Authors only. Freq, Frequence of Total Publications. MCP_Ratio, Proportion of Multiple Country Publications. TP, Total Publications. TP_rank, Rank of Total Publications. TC, Total Citations. TC_rank, Rank of Total Citations. Average Citations, The average number of citations per publication.

### Analysis of institutions

**Figure 4:**
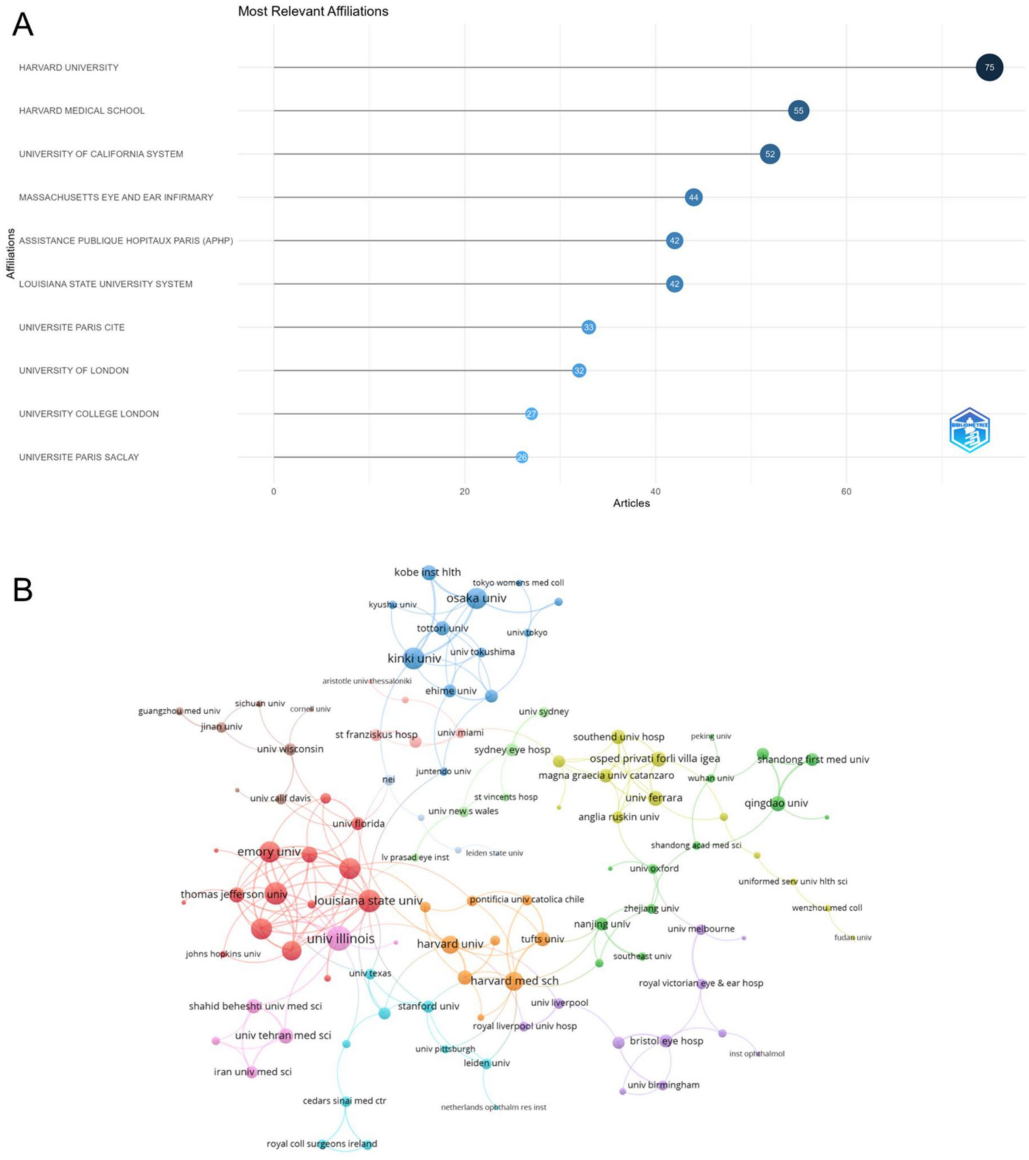
Analysis of institutions. **(A)** Top 10 institutions ranked by article count, obtained using the Bibliometrix package in R 4.3.3. **(B)** Visualization map illustrating collaboration among different institutions using VOSviewer.

The top 10 institutions, ranked by the number of articles published in the field, are listed in Figure 4A. Harvard University ranks first with 75 articles, followed by Harvard Medical School with 55 articles, and the University of California System with 52 articles. Other notable institutions include Massachusetts Eye and Ear Infirmary and Assistance Publique Hopitaux Paris, contributing 44 and 42 articles, respectively. The collaboration network is presented in Figure 4B, highlighting institutions such as Harvard University and the Louisiana State University System as prominent central nodes. The red cluster reflects strong collaborative networks among U.S. institutions, with key connections among Emory University, the University of Florida, and the University of Illinois. The green and blue clusters reflect collaborations primarily among European and Asian institutions. Among the 147 institutions engaged in international collaborations with at least three articles, the University of Illinois led with 25 collaborations, followed by Louisiana State University (22) and Baylor College of Medicine (21).

### Analysis of journals

**Figure 5:**
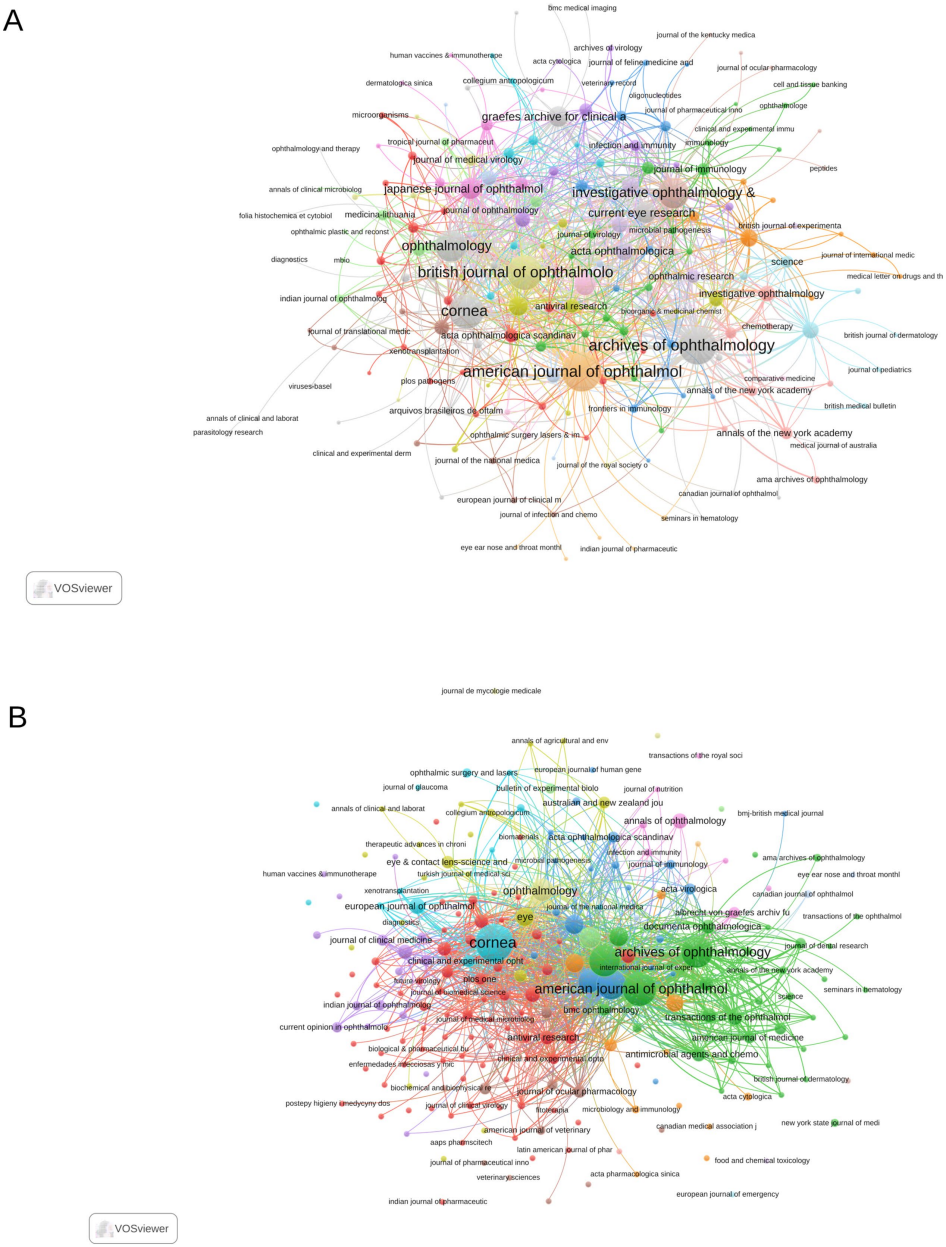
Analysis of journals using VOSviewer. **(A)** Co-occurrence network of journals. **(B)** Coupling network of journals.

Several bibliometric indicators for the top 20 most productive journals in research on “Herpes Simplex Keratitis” from 1941 to 2024 are provided in Table 2. *American Journal of Ophthalmology* ranks first with the highest h-index of 29 and a total of 82 publications, alongside the highest total citation count of 1,636. *Cornea* follows closely, with an h-index of 26, 105 publications, and 1,362 citations. *Archives of Ophthalmology* holds third place with 71 publications, an h-index of 28, and 1,481 citations. The top three journals with the highest total link strength in co-occurrence networks are the *American Journal of Ophthalmology* (465), *Archives of Ophthalmology* (435), and *Cornea* (355) (Figure 5A). The coupling networks of journals also include 20 journals with at least three couples, with the highest total link strength observed in *Cornea* (7,125), *Investigative Ophthalmology & Visual Science* (5,531), and *British Journal of Ophthalmology* (5,458) (Figure 5B).

**Table 2:** Bibliometric indicators of high-impact journals.

Source	H_index	JCR Quartile_2023	IF_2023	TC	TC_RANK	TP	TP_RANK	PY_start
American Journal of Ophthalmology	29	1	4.1	1,636	1	82	2	1948
Archives of Ophthalmology*	28	NA	NA	1,481	2	71	3	1941
Cornea	26	2	1.9	1,362	3	105	1	1991
Investigative Ophthalmology & Visual Science	25	1	5	1,248	5	60	5	1977
Ophthalmology	24	1	13.1	1,255	4	28	7	1982
British Journal of Ophthalmology	22	1	3.7	997	6	64	4	1964
Current Eye Research	13	3	1.7	347	10	30	6	1981
Experimental Eye Research	11	1	3	223	20	17	11	1963
Japanese Journal of Ophthalmology	11	2	2.1	134	33	24	8	1980
Eye	10	1	2.8	256	13	17	12	1988
Graefes Archive for Clinical and Experimental Ophthalmology	10	2	2.4	152	28	19	10	1983
Acta Ophthalmologica	9	1	3	15	192	19	9	1967
Journal of Cataract and Refractive Surgery	8	1	2.6	234	18	10	20	1995
Antimicrobial Agents and Chemotherapy	7	1	4.1	252	15	8	22	1975
Clinical and Experimental Ophthalmology	7	1	4.9	103	38	7	27	2000
Investigative Ophthalmology	7	1	5	121	36	10	19	1962
Journal of Infectious Diseases	7	1	5	354	9	7	29	1976
Journal of Medical Virology	7	1	6.8	41	82	7	30	1987
Ophthalmologica	7	2	2.1	57	58	14	13	1975
Transactions of the Ophthalmological Societies of the United Kingdom*	7	NA	NA	86	45	11	17	1973

H_index, The h-index of the journal, which measures both the productivity and citation impact of the publications. IF, Impact Factor, indicating the average number of citations to recent articles published in the journal. JCR_Quartile, The quartile ranking of the journal in the Journal Citation Reports, indicating the journal’s ranking relative to others in the same field (Q1: top 25%, Q2: 25–50%, Q3: 50–75%, Q4: bottom 25%). TP, Total Publications. TP_rank, Rank of Total Publications. TC, Total Citations. TC_rank, Rank of Total Citations. Average Citations: The average number of citations per publication. PY_start, Publication Year Start, indicating the year the journal started publication. *Not currently included in the SCI-Expanded and SSCI journal listings.

### Analysis of authors

**Figure 6:**
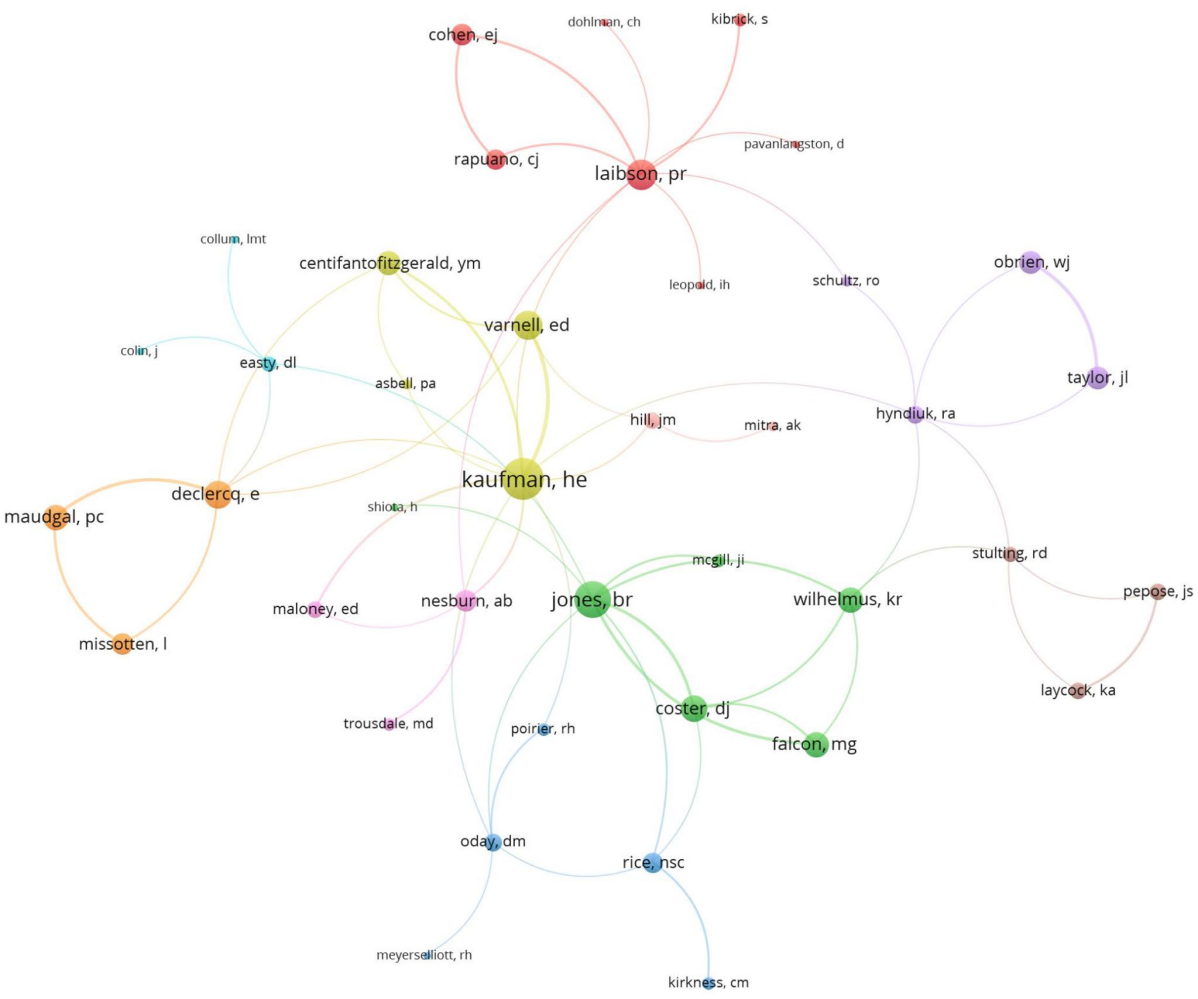
Visualization map depicting the collaboration among different authors (VOSviewer).

A total of 3,867 authors contributed to publications on HSK. The 20 most influential authors accounted for 262 articles, with a total of 8,626 citations. Kaufman, HE was the most cited author (1,988 citations, h-index = 24, TP = 40), followed by Coster, DJ (763 citations, h-index = 9, TP = 10) and Laibson, PR (543 citations, h-index = 12, TP = 14) (Table 3). Kaufman, HE played a central role in the collaboration network, showing strong collaborations with authors like Jones, BR, and Coster, DJ. Among the 115 authors involved in international collaborations with at least four articles, Shimomura, Y had the most international partnerships (45), followed by Inoue, Y (43) and Kaufman, HE (30) (Figure 6).

**Table 3:** Publication and citation profiles of high-impact authors.

Author	h_index	g_index	m_index	PY_start	TP	TP_RANK	TP_FRAC	TC	TC_RANK
Kaufman HE	24	40	0.375	1961	40	1	15.4	1988	1
Jones BR	13	16	0.245	1972	16	3	4.63	514	5
Laibson PR	12	14	0.194	1963	14	7	3.52	543	3
Inoue Y	11	16	0.306	1989	16	3	2.48	265	14
Pavanlangston D	11	11	0.22	1975	11	9	3.56	339	10
Foster CS	10	16	0.227	1981	16	3	4.36	372	8
Nesburn AB	10	10	0.159	1962	10	12	2.62	410	6
Shimomura Y	10	14	0.263	1987	14	7	2.25	255	16
Wilhelmus KR	10	11	0.222	1980	11	9	3.13	401	7
Coster DJ	9	10	0.188	1977	10	12	3.35	763	2
Declercq E	9	10	0.2	1980	10	12	2.68	267	13
Easty DL	9	15	0.173	1973	15	6	4.58	248	17
Okumoto M	9	16	0.136	1959	18	2	5.37	277	12
Varnell ED	9	10	0.184	1976	10	12	2.73	246	18
Maudgal PC	8	9	0.17	1978	9	16	3.08	256	15
Stulting RD	8	8	0.2	1985	8	18	1.71	335	11
Cohen EJ	7	7	0.167	1983	7	19	1.11	358	9
Hamrah Pedram	7	11	0.467	2010	11	9	1.55	516	4
Kimura SJ	7	7	0.101	1956	7	19	2.75	171	19
Ohashi Y	7	9	0.156	1980	9	16	2.57	102	20

H_index, The h-index of the journal, which measures both the productivity and citation impact of the publications. g_index, The g-index of the journal, which gives more weight to highly-cited articles. m_index, The m-index of the journal, which is the h-index divided by the number of years since the first published paper. TP, Total Publications. TP_rank, Rank of Total Publications. TC, Total Citations. TC_rank, Rank of Total Citations. Average Citations, The average number of citations per publication. PY_start, Publication Year Start, indicating the year the journal started publication.

### Analysis of keywords

**Figure 7:**
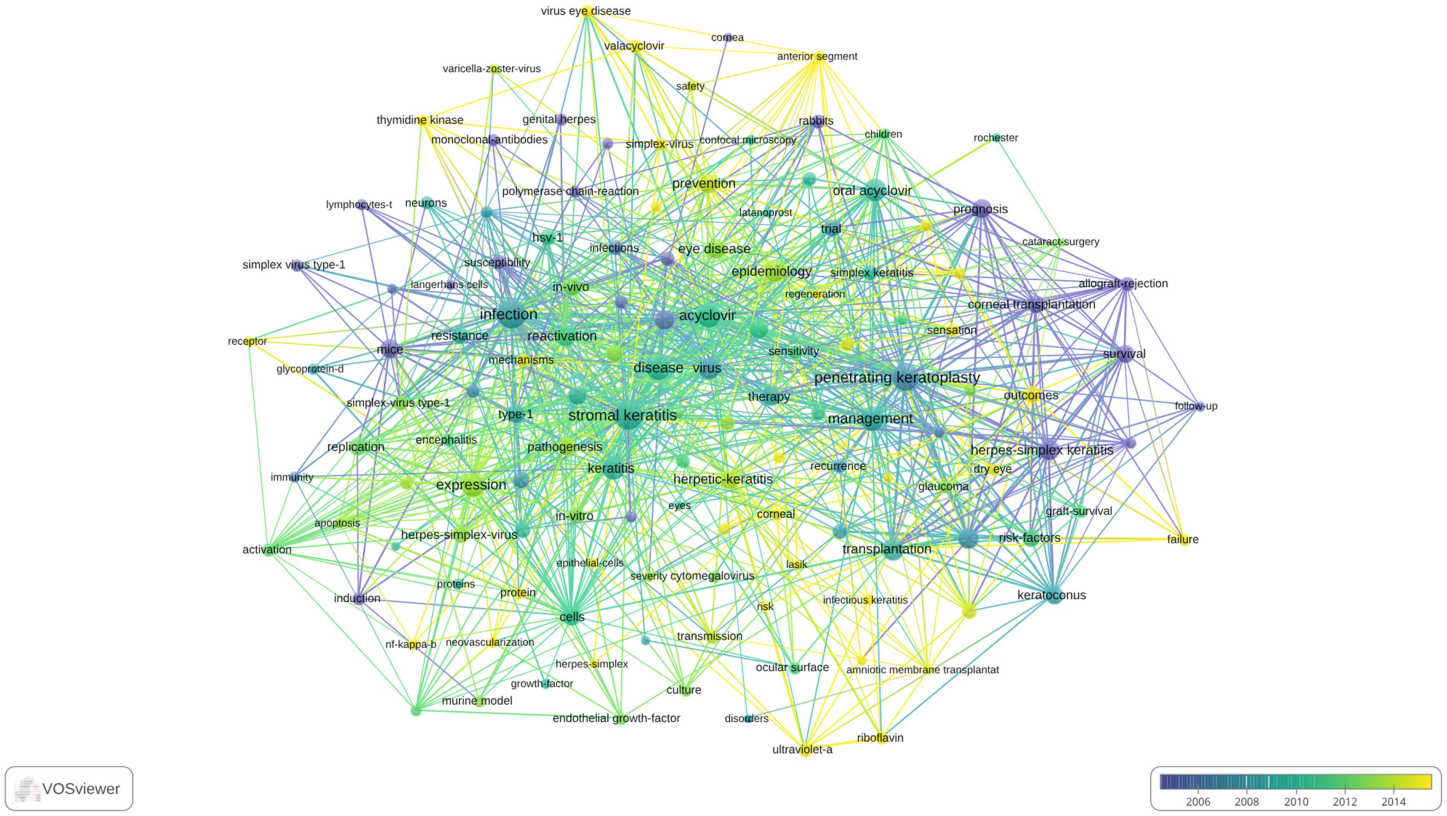
Visual analysis of keyword co-occurrence network (VOSviewer).

A total of 1,194 keywords were extracted from the 1,076 articles, and keywords with an occurrence of 20 or more were analyzed. The visualization of the analysis is presented in Figure 7. The most frequently occurring keyword was “infection” (82 occurrences), followed by “stromal keratitis” (73 occurrences) and “penetrating keratoplasty” (62 occurrences). In terms of total link strength, “infection” and “stromal keratitis” were the highest (both with a total link strength of 311), followed by “penetrating keratoplasty” (259) and “disease” (198).

### Analysis of burst keywords

**Figure 8:**
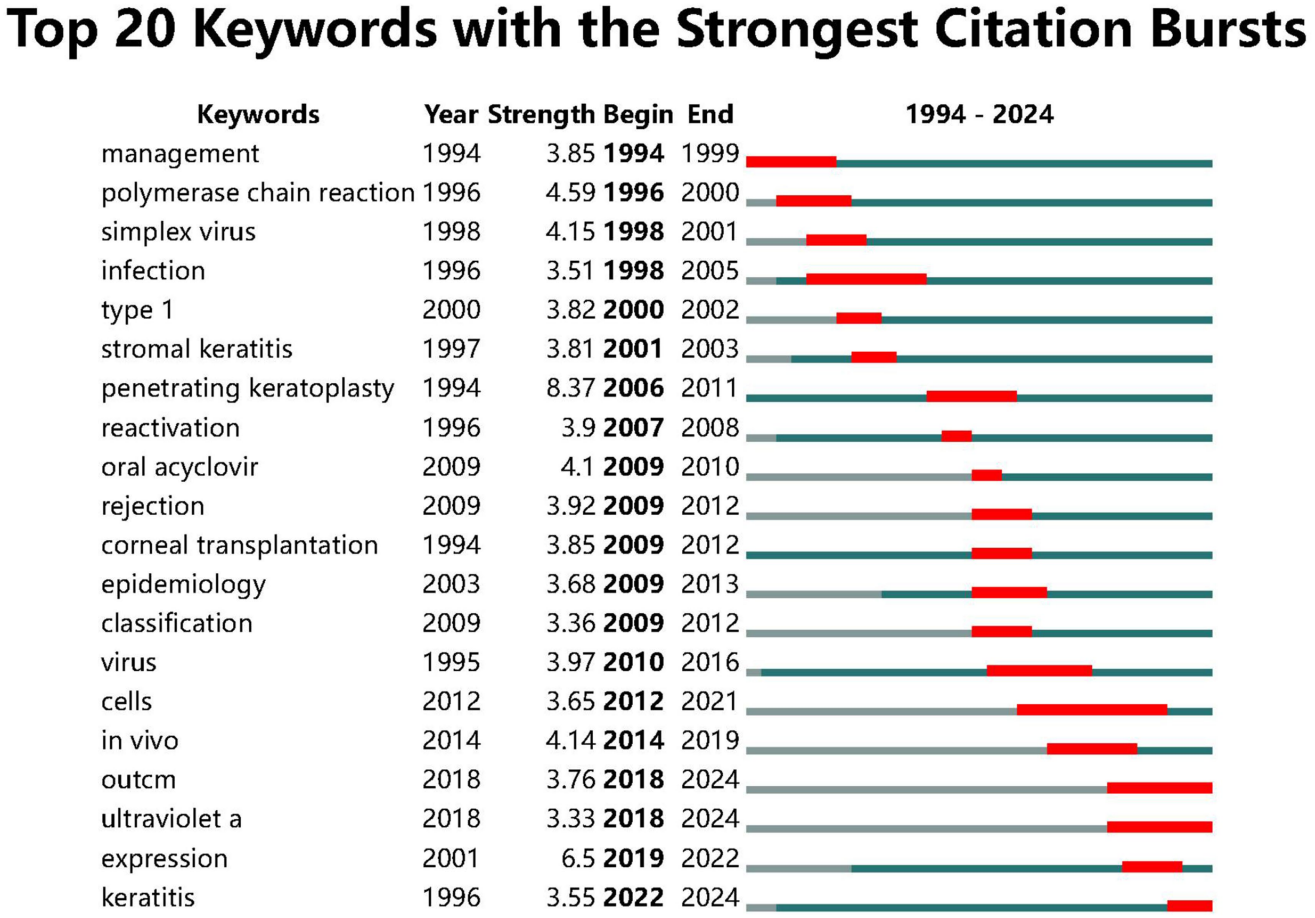
Top 20 keywords with the strongest citation bursts (CiteSpace).

Keyword burst analysis revealed that earlier terms like “management,” “polymerase chain reaction,” and “simplex virus” experienced significant bursts between 1994 and 2000. Between 2006 and 2012, keywords such as “penetrating keratoplasty,” “reactivation,” and “rejection” became more prominent, marking critical phases in research on corneal transplantation and related complications. More recent terms, including “outcome,” “ultraviolet A,” and “keratitis,” have gained prominence since 2018, highlighting future research trends (Figure 8).

## Discussion

The annual publication output in HSK research has shown a steady increase since the 2000s, peaking in 2023 with 40 articles, indicating growing academic interest. The United States leads the field with 267 articles, followed by China and Japan. The USA ranks first in both total publications and citations, reflecting its significant research impact. Institutions such as Harvard University and the University of California System show strong research productivity. Despite high output, countries like the Netherlands and Australia have demonstrated a higher average citation per article, highlighting their influential research contributions. In terms of journal impact, *American Journal of Ophthalmology* and *Cornea* are notable for their significant contributions, with *American Journal of Ophthalmology* ranking first in the h-index, while *Cornea* has the highest number of publications. These journals play central roles in both co-citation and coupling networks, underscoring their influence in shaping research trends in HSK. Kaufman H E emerged as the most cited author, with 1,988 citations. His studies are primarily published in high-quality journals such as *Ophthalmology* (21), *American Journal of Ophthalmology* (22) and *Current Eye Research* (23). The author’s main research focus is on evaluating the safety and efficacy of various treatments, including ganciclovir ophthalmic gel and topical corticosteroids, for HSK and exploring alternative therapeutic options to improve patient outcomes.

### Research topics and frontiers in HSK

Ultraviolet A (UVA) therapy, combined with riboflavin, has emerged as a promising treatment method, particularly for its ability to strengthen corneal collagen cross-linking and inhibit viral replication (24). This approach has been highlighted as an innovative way to address the challenges of recurrent HSK. Future studies could explore optimizing the parameters of UVA therapy, such as the wavelength, duration, and intensity of light exposure, to maximize its therapeutic effects while minimizing potential side effects, such as phototoxicity or damage to surrounding tissues. Additionally, research should investigate whether combining UVA therapy with existing antiviral agents, such as ganciclovir or valacyclovir, could yield synergistic effects to enhance treatment efficacy and reduce recurrence rates (25).

Beyond UVA therapy, other emerging approaches warrant further exploration. For instance, nanoparticle-based drug delivery systems have shown potential in delivering antiviral medications or immunomodulatory agents directly to the cornea, improving both drug bioavailability and patient outcomes (26). Similarly, gene therapy targeting the reactivation pathways of herpes simplex virus (HSV) could provide long-term solutions to prevent recurrent episodes of HSK (27). Developing these advanced therapeutic strategies could transform the clinical management of HSK, offering patients more effective and personalized treatment options.

Furthermore, the role of immunomodulatory therapies should be emphasized in future studies. As identified, immune rejection and inflammation play critical roles in HSK recurrence (28). Novel immunosuppressive agents or biologics, such as monoclonal antibodies targeting specific inflammatory pathways, could help regulate immune responses and prevent corneal scarring. For example, therapies that expand regulatory T cells or inhibit effector T cells involved in keratitis progression could mitigate the chronic inflammation associated with HSK (29).

Keywords (mainly from research around 2010) include “infection,” “stromal keratitis,” and “management.” Research during this period focused on the clinical management and treatment strategies of HSK, particularly on how to effectively control stromal keratitis infections to prevent the disease from progressing to more severe corneal damage or blindness. For example, Gustavo Zapata and colleagues (30), through a comparative study in a mouse model of herpetic stromal keratitis, found that topical application of 0.05% rapamycin was more effective than 0.5% cyclosporine and showed similar efficacy to 0.1% dexamethasone in minimizing the immuno-inflammatory process, and also demonstrated early inhibition of new vessel formation. A study on the combination therapy of TNFRSF25 agonistic antibody and galectin-9 in a mouse model of herpetic stromal keratitis found that this combined approach effectively controlled immunoinflammatory lesions more than monotherapy, with the beneficial outcome attributed to the expansion of regulatory T cells and reduction of effector T cells responsible for tissue damage (31).

Keywords (representing the latest research from 2014 onwards) include “rejection,” “reactivation,” and “prevention.” Recent research has shifted toward the mechanisms of HSK recurrence, with a particular focus on preventing disease recurrence and controlling immune rejection, aiming to reduce recurrence rates and improve treatment outcomes. Hongmin Yun and colleagues, through a study on mice with herpes simplex stromal keratitis, found that much of the corneal inflammation associated with the disease is attributable to damage to corneal nerves and the accompanying loss of blink reflex, which can be prevented or ameliorated by tarsorrhaphy, and that this nerve damage and loss of blink reflex are reversible and regulated by CD4 (+) T cells (32). A study (33) has concluded that damage to corneal sensory nerves and the associated loss of blink reflex can exacerbate and prolong inflammation-induced pathology in mice with HSK. Preventing corneal desiccation results in a milder and more transient HSK with variable scarring that mirrors HSK seen in most humans. Additionally, the study found that nerve damage is reversible and regulated by CD4 (+) T cells. Devin M West and colleagues (34), through a study on the role of IL-6 and CXCL1 in recurrent herpetic stromal keratitis, found that CXCL1 is required for the disease to recur, while IL-6 does not play a role in recurrent HSK. Based on burst keyword analysis, future research trends show a strong focus on treatment and patient clinical outcomes. Since 2018, keywords such as “outcome” and “ultraviolet A” have exhibited significant research growth. It is expected that future studies will focus on how innovative treatments, such as ultraviolet A therapy, can be used to control HSK and further optimize clinical outcomes.

### Strengths and limitations

This bibliometric investigation provides a comprehensive exploration of the distribution trends and key research focuses in HSK. One of the major strengths of this study is its multi-year analysis, aimed at identifying relevant literature in the field. However, like previous bibliometric studies, several limitations exist. First, potential biases include the reliance on citation counts, which may not fully capture an article’s clinical impact. Additionally, the exclusion of non-English publications could limit the scope of the analysis. The dependence on specific databases might have led to the exclusion of relevant literature not indexed in these sources. Moreover, while bibliometric methods offer valuable insights, they have limitations in capturing the full scope and nuances of a research field.

This bibliometric analysis indicates that the primary research hotspots in HSK currently center around two main areas: the clinical management and treatment strategies for controlling stromal keratitis and infection, and the mechanisms of HSK recurrence, with a focus on preventing reactivation and managing immune rejection. Looking ahead, the future research trajectory appears to be increasingly focused on innovative treatment methods, such as ultraviolet A therapy, nanoparticle-based drug delivery, and advanced immunomodulatory approaches, to control HSK and improve patient clinical outcomes.

## Data Availability

The original contributions presented in the study are included in the article/supplementary material, further inquiries can be directed to the corresponding author.
